# Association of biofilm formation, antimicrobial resistance, clinical characteristics, and clinical outcomes among *Acinetobacter baumannii* isolates from patients with ventilator‐associated pneumonia

**DOI:** 10.1111/crj.13732

**Published:** 2024-01-12

**Authors:** Arnon Chukamnerd, Niwat Saipetch, Kamonnut Singkhamanan, Natnicha Ingviya, Nawaporn Assanangkornchai, Komwit Surachat, Sarunyou Chusri

**Affiliations:** ^1^ Division of Infectious Diseases, Department of Internal Medicine, Faculty of Medicine Prince of Songkla University Hat Yai Thailand; ^2^ Department of Biomedical Sciences and Biomedical Engineering, Faculty of Medicine Prince of Songkla University Hat Yai Thailand; ^3^ Department of Pathology, Faculty of Medicine Prince of Songkla University Hat Yai Thailand; ^4^ Translational Medicine Research Center, Faculty of Medicine Prince of Songkla University Hat Yai Thailand

**Keywords:** *Acinetobacter baumannii*, antimicrobial resistance, biofilm formation, ventilator‐associated pneumonia

## Abstract

**Introduction:**

Biofilm formation is an important virulence factor of *Acinetobacter baumannii* . Here, we examined the biofilm formation of archived *A. baumannii* causing ventilator‐associated pneumonia (VAP).

**Methods:**

Eighteen and twenty isolates of *A. baumannii* causing bacteremic pneumonia and non‐bacteremic pneumonia were included, respectively. Antimicrobial susceptibility testing was performed by broth microdilution method, while biofilm formation was evaluated by microtiter dish biofilm formation assay.

**Results:**

All 38 isolates were still susceptible to colistin and tigecycline, whereas almost all isolates were non‐susceptible (intermediate to resistant) to several antimicrobial agents, especially ceftriaxone and cefotaxime. Approximately, 44% of bacteremic isolates and 50% of non‐bacteremic isolates were classified as carbapenem‐resistant *A. baumannii* (CRAB). Biofilm formation was detected in 42% of the studied isolates. Bacteremia among the patients infected with biofilm‐producing isolates was significantly higher than in those infected with non‐biofilm‐producing isolates. The antimicrobial susceptibilities of *A. baumannii* with biofilm formation were lower than those without biofilm formation, but the differences did not have statistical significance. The patients infected with non‐biofilm‐producing isolates had good clinical and non‐clinical outcomes than those infected with biofilm‐producing isolates. The survival rate of patients diagnosed with VAP due to biofilm‐producing *A. baumannii* was lower than in those patients diagnosed with VAP due to non‐biofilm‐producing isolates.

**Conclusion:**

Biofilm formation of *A. baumannii* causing VAP was associated with antimicrobial resistance and bacteremia as well as unfavorable clinical outcomes.

## Graphical abstract

Biofilm formation was associated with increased antimicrobial resistance, higher incidence of bacteremia, and unfavorable clinical outcomes. The patients infected with non‐biofilm‐producing isolates had better outcomes compared to those infected with biofilm‐producing isolates. This study highlights the importance of biofilm formation as a virulence factor in *A. baumannii* infections and its impact on patient outcomes with VAP.

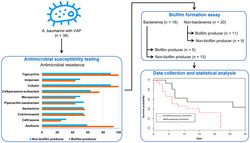

## 1 INTRODUCTION

*Acinetobacter baumannii* is one of the most important pathogens causing nosocomial infection worldwide.^1^ With its propensity for multidrug resistance, infections caused by this pathogen have unfavorable outcomes and cause high economic burdens.^2^, ^3^ *A. baumannii* is mostly involved in healthcare‐related respiratory infections, particularly ventilator‐associated pneumonia (VAP), bloodstream infections (BSIs), and surgical wound infections (SWIs).^4^ Several risk factors increasing the mortality and morbidity of *A. baumannii* infections (especially carbapenem‐resistant strains) have been documented, including exposure to various antimicrobial agents, receiving medical devices, and colonization with this pathogen.^5^ However, the emergence of carbapenem‐resistant *A. baumannii* (CRAB) without those established risk factors was also reported. CRAB pneumonia causes a prolonged length of hospital stay and high costs of healthcare with a relatively substantial mortality rate, while CRAB bacteremia was significantly associated with a high mortality rate.^6^, ^7^ The predictors for CRAB bloodstream infection among pneumonic patients are still unknown. Although the patients were treated with antimicrobial agents providing good in vitro activity against CRAB, their clinical outcomes were still poor.^7^ The potential explanations for the unexpected emergence and unsuccessful treatment of CRAB have been focused on the intrinsic virulent properties of this pathogen.^8^

The mechanisms of disease caused by *A. baumannii* are in combination with bacterial virulence factors, nutrient acquisition, community interactions, and genetic regulation of virulence phenotypes.^9^ Community interactions between neighboring cells by quorum sensing are the key to the success of the *A. baumannii* population. AHL‐dependent quorum sensing is necessary for biofilm development in this pathogen.^10^ Biofilm‐forming processes are composed of three steps, the initial attachment, biofilm aggregation into a small colony, maturation, and biofilm dispersal into the environment.^11^ This increased virulence arises from the ability of the organism to create a bacterial cell film that attaches to the surfaces of human or non‐human structures and to contact other bacterial cells to contribute to the specific configuration of the community.^12^ Biofilm formation plays an important role in antibiotic resistance through low cell permeability, efflux pumps, and modifying enzymes.^13^ These antibiotic resistance mechanisms provided by biofilm lead to ineffective eradication of the bacteria.

The main concern of *A. baumannii* biofilms is that they are greatly tolerant to antibiotics and can potentially develop antimicrobial resistance.^13^ Additionally, biofilm formation assists bacteria to survive in the bloodstream by raising the number of bacteria and protecting them from human immunity.^14^ However, the association between biofilm formation, antimicrobial resistance, clinical characteristics, and clinical outcomes of *A. baumannii* has not been broadly studied. The purpose of this study was to integrate clinical data, including characteristics of the infections, clinical outcomes, and antimicrobial susceptibility of *A. baumannii* isolates with an in vitro study of biofilm formation among pneumonic patients.

## 2 MATERIALS AND METHODS

### 2.1 Patients and setting

The study was conducted in the intensive care units (ICUs) of Songklanagarind Hospital, a university hospital and medical referral center in Southern Thailand. Two ICUs with 30 admission beds (12 beds in the medical ICU and 18 beds in the surgical ICU) and approximately 9000 patient‐admission days per year were included in the study. The sputum suctions (respiratory clinical samples) were obtained from adult (age ≥18 years) patients who were admitted from January 1, 2012, to December 31, 2012, and diagnosed with *A. baumannii* pneumonia were enrolled in the study. VAP was defined as a new and persistent infiltrate on chest radiograph plus two or more of the three criteria namely fever of >38.3°C, leukocytosis of >12 × 10^9^/mL, and/or purulent tracheobronchial secretions after 48 h of intubation and mechanical ventilation.^15^ Only the first episodes of pneumonia were enrolled in the analysis to avoid case duplication. Patients with concomitant pulmonary infection with other organisms were excluded from the study.

### 2.2 Bacterial identification

*A. baumannii* complexes were identified as oxidase‐negative, non‐motile, lactose‐nonfermenting Gram‐negative coccobacilli with standard biochemical testing in the clinical microbiology laboratory. The presence of the *bla*_OXA‐51‐like_ gene was detected in all studied isolates, using the Polymerase Chain Reaction (PCR) method with primers F_oxa51_001 (5′‐TAATGCTTTGATCGGCCTTG‐3′) and R_oxa51_001 (5′‐TGGATTGCACTTCATCTTGG‐3′). Isolates with a positive result of *bla*_OXA‐51‐like_ gene were assigned as *A. baumannii*, while isolates with a negative result of *bla*_OXA‐51‐like_ gene proceeded to *rpoB* gene sequencing. For the suspected *A. baumannii* isolates, the species was confirmed by Matrix‐Assisted Laser Desorption Ionization‐Time of Flight Mass Spectrometry (MALDI‐TOF/MS) (Bruker Daltonics, Bremen, Germany) with ClinPro Tools software (version 2.2; Bruker Community‐Acquired *A. baumannii bacteremia* 797 Daltonics). Meanwhile, the presence of *rpoB* gene was investigated in the *bla*_OXA‐51‐like_‐negative isolates (non‐suspected *A. baumannii* isolates), using primers rpoB‐F (5′‐TAYCGYAAAGAYTTGAAAGAAG‐3′) and rpoB‐R (5′‐CMACACCYTTGTTMCCRTGA‐3′), and then the PCR products of *rpoB* gene was sequenced.^16^ Nucleotide sequence homology searches of the *rpoB* gene sequences were analyzed using the Basic Local Alignment Search Tool (BLAST) (https://www.ncbi.nlm.nih.gov/), compared with the previously published *rpoB* sequences of the *Acinetobacter* species.^17^

### 2.3 Antimicrobial susceptibility testing

Minimum inhibitory concentrations (MICs) of 15 antimicrobial agents, including imipenem, meropenem, ampicillin‐sulbactam, cefoperazone‐sulbactam, piperacillin‐tazobactam, ceftriaxone, cefotaxime, ceftazidime, gentamicin, amikacin, ciprofloxacin, levofloxacin, trimethoprim‐sulfamethoxazole, colistin, and tigecycline, were evaluated using the broth microdilution method, and the results were interpreted, according to the Clinical and Laboratory Standards Institute (CLSI) guidelines^18^ and a previous study.^19^ The isolates with the MICs of imipenem or meropenem of > 2 μg/mL were defined as carbapenem‐resistant *A. baumannii* (CRAB).

### 2.4 Biofilm formation assay

The microtiter dish assay described by O'Toole GA was conducted in the study of biofilm formation.^20^ *A. baumannii* isolates were cultured in trypticase soy broth (TSB) at 37°C with 180 rpm for 18 h, and then they were diluted 1:1 into TSB for biofilm assays. One hundred microlitres of dilution was added to a 96‐well plate with three replicates for each treatment and incubated at 37°C for 18 h. A well without bacterial suspension and antibiotic treatment was defined as a negative control. The planktonic growth was removed by phosphate‐buffered saline (PBS), and then 0.1% crystal violet was added to stain the biofilm and incubated for 15 min. The well plates were rinsed with sterile distilled water and dried. After that, 95% ethanol was added to solubilize them into crystal violet for 10 min. Biofilm formation was quantified by OD_570_ spectrophotometry. The wells with an OD_570_ value more than ODc (ODc = 3SD mean of negative control + average OD of negative control) were considered as positive biofilm formation. Differences in optical density levels were also used to interpret quantitative measurements of biofilm formation. The experiment was done in triplicate, and *A. baumannii* ATCC 19606 was used as a positive control for the accuracy of biofilm formation.

### 2.5 Data collection

The medical records of all studied patients were reviewed. Information received from the medical record included sex, age, underlying medical condition, or comorbidities. The comorbidities included diabetes mellitus, cerebrovascular disease, cardiovascular disease (myocardial infarction or heart failure from any causes), and immunocompromised status (hematologic malignancy, solid organ malignancy, HIV infection). Additionally, Acute Physiology and Chronic Health Evaluation (APACHE) II score; indwelling intravascular devices; having received during current admission or currently receiving an invasive procedure such as bronchoscopy, gastroscopy, or tissue biopsy from visceral organs, endotracheal tube, urinary catheter, and other types of tube drainage; and prior antimicrobial exposure were also used. The outcomes included 14‐day and 30‐day mortality, in‐hospital mortality, hospital costs, and length of hospital stay. The lengths of hospital stays were separated into total lengths of stay and lengths of stay after identification of infection. Hospital costs were categorized into antimicrobial costs and non‐antimicrobial costs.

### 2.6 Statistical analysis

The demographic data, clinical characteristics, and outcomes were analyzed and compared by Student's *t* test for continuous variables and the chi‐square test or Fisher's exact test for categorical variables. The variable differences were analyzed with a 95% confidence interval (CI) and odds ratio (OR). Associations with ORs for 30‐day mortality were recognized by the chi‐square test or Fisher's exact test. The variables with a *p*‐value of 0.2 were incorporated in multivariate logistic regression models which were used to evaluate the effect of each characteristic, presented as adjusted ORs. All independent variables were incorporated into the final model. The association of each variable with the outcome was illustrated with adjusted ORs and 95% CIs. The significance level was set at 0.05. Survival analysis and Cox proportional hazard regression were used to compare the differences in duration of survival between the biofilm formation and non‐biofilm formation groups. The starting time for the different analyses was set as the date of infection and the ending time was defined as the date of outcome was registered.

## 3 RESULTS

### 3.1 Clinical characteristics of the patients

In 2012, a total of 38 patients diagnosed with VAP due to *A. baumannii* were admitted to the ICUs of Songklanagarind Hospital. The clinical characteristics are exhibited in Table 1. Comorbidities in these patients included diabetes mellitus, cardiovascular disease, chronic kidney disease, cerebrovascular disease, and immunocompromised status (solid organ malignancy, hematologic malignancy, and HIV infection). Importantly, the patients previously received antimicrobial therapy, including cephalosporins, carbapenems, fluoroquinolones, and other antibiotics. In addition, 18/38 (47%) patients were concomitated with bacteremia, while 20/38 (53%) patients were not concomitated with bacteremia.

**TABLE 1:** Clinical characteristics of the study patients with ventilator‐associated pneumonia due to *A. baumannii* .

Clinical characteristic	Value (%) for patients (*N* = 38)
Median [IQR] of patient's age (years)	64 [47, 71]
Male sex	22 (58%)
Female sex	16 (42%)
Comorbidities	
Diabetes mellitus	11 (29%)
Cardiovascular disease	9 (24%)
Chronic kidney disease	9 (24%)
Cerebrovascular disease	4 (10%)
Immunocompromised status	
Solid organ malignancy	11 (29%)
Hematologic malignancy	4 (10%)
HIV infection	2 (5%)
Initial admission to intensive care unit	28 (74%)
Median [IQR] of duration (days) from admission to infection	6 [4, 9]
Median [IQR] of APACHE II score	20 [15, 22]
Retained medical devices	
Urinary catheterization	34 (89%)
Multiple devices	33 (87%)
Intravascular device	31 (82%)
Receiving invasive procedure	18 (47%)
Previous antimicrobial therapy	
Cephalosporin (s)	14 (37%)
Carbapenem (s)	10 (26%)
Fluoroquinolone (s)	2 (5%)
Other	6 (16%)
Concomitant bacteremia	18 (47%)

Abbreviations: APACHE II, Acute Physiology and Chronic Health Evaluation; HIV, human immunodeficiency virus; IQR, interquartile range.

### 3.2 Biofilm formation and antimicrobial susceptibility patterns

In 38 *A. baumannii* isolates, 16 (42%) and 22 (58%) isolates were classified as biofilm‐producing and non‐biofilm‐producing *A. baumannii*, respectively (Table 2). Only concomitant bacteremia among the patients infected with biofilm‐producing *A. bauamnnii* isolates was significantly higher than in those infected with non‐biofilm‐producing isolates (*p* = 0.028). The results of antimicrobial susceptibility in all studied isolates are illustrated in Table 3. We found that the high numbers of *A. bauamnnii* with and without biofilm formation were non‐susceptible (intermediate to resistant) to almost all tested antimicrobial agents, while all of the isolates were still susceptible to colistin and tigecycline. Moreover, 8/18 (44%) bacteremic isolates and 10/20 (50%) isolates non‐bacteremic isolates were also classified as carbapenem‐resistant *A. baumannii* (CRAB). The antimicrobial‐susceptible *A. bauamnnii* isolates with biofilm formation were slightly lower than those without biofilm formation but the differences did not reach statistical significance, except for cefoperazone‐sulbactam (*p* = 0.042).

**TABLE 2:** Comparisons of clinical characteristics between the study patients with ventilator‐associated pneumonia caused by biofilm‐producing *A. baumannii* and those with ventilator‐associated pneumonia caused by non‐biofilm‐producing *A. baumannii* .

Parameter	Values (%) of patients with ventilator‐associated pneumonia due to		*p‐*value
	Biofilm‐producing *A. baumannii* (*n* = 16)	Non‐biofilm‐producing *A. baumannii* (*n* = 22)	
Demographic data			
Median [IQR] of patient's age (years)	62 [48, 73]	60 [41, 71]	0.856
Male sex	10 (62%)	12 (54%)	0.624
Comorbidities	16 (100%)	20 (91%)	0.379
Clinical characteristics			
Emergency indication for admission	12 (75%)	18 (82%)	0.612
Starting admission to intensive care unit	12 (75%)	16 (73%)	0.875
Duration (days) from admission to infection, median (IQR)	7 (4, 8)	6 (4, 8)	0.752
Median [IQR] of APACHE II score	21 [15, 24]	20 [14, 24]	0.802
Use of medical devices (including mechanical ventilator)	16 (100%)	19 (86%)	0.251
Received invasive procedure (s)	8 (50%)	10 (45%)	0.782
Concomitant bacteremia	11 (69%)	7 (32%)	0.028

Abbreviations: APACHE II, Acute Physiology and Chronic Health Evaluation; IQR, interquartile range.

**TABLE 3:** Comparisons of antimicrobial susceptibilities between biofilm‐producing *A. baumannii* and non‐biofilm‐producing *A. baumannii* isolates.

Antimicrobial agent (s)	No. (%) of susceptible isolates		*p*‐value
	Biofilm‐producing *A. baumannii* (*n* = 16)	Non‐biofilm‐producing *A. baumannii* (*n* = 22)	
Imipenem	5 (31%)	13 (59%)	0.095
Meropenem	5 (31%)	13 (59%)	0.095
Ampicillin‐sulbactam	2 (12%)	9 (41%)	0.070
Cefoperazone‐sulbactam	4 (25%)	13 (59%)	0.042
Piperacillin‐tazobactam	3 (19%)	11 (50%)	0.056
Ceftriaxone	1 (6%)	3 (14%)	0.474
Cefotaxime	1 (6%)	3 (14%)	0.474
Ceftazidime	4 (25%)	11 (50%)	0.126
Gentamicin	5 (31%)	8 (36%)	0.743
Amikacin	5 (31%)	9 (41%)	0.543
Ciprofloxacin	3 (19%)	10 (45%)	0.096
Levofloxacin	3 (19%)	11 (50%)	0.057
Trimethoprim‐sulfamethoxazole	2 (12%)	7 (32%)	0.180
Colistin	16 (100%)	22 (100%)	0.999
Tigecycline	16 (100%)	22 (100%)	0.999

In addition, we have reported the MIC_50_ and MIC_90_ values of all tested antimicrobial agents against both *A. baumannii* isolates with and without biofilm formation, as shown in Table 4. Notably, the MIC_50_ and MIC_90_ values of imipenem, meropenem, colistin, and tigecycline consistently remained low at 1 μg/mL, regardless of whether the *A. baumannii* isolates exhibited biofilm formation. Conversely, aminoglycosides (gentamicin and amikacin) demonstrated higher MIC_50_ and MIC_90_ values (>256 μg/mL) against both types of *A. baumannii*. When comparing the MIC values between these two *A. baumannii* types, we observed that the MIC_50_ values of ampicillin‐sulbactam, cefoperazone‐sulbactam, piperacillin‐tazobactam, ceftazidime, ciprofloxacin, and levofloxacin against biofilm‐producing isolates were slightly higher than those against non‐biofilm‐producing isolates. It is noteworthy that among these antibiotics, only the MIC_90_ value of ampicillin‐sulbactam against biofilm‐producing isolates showed a similar trend of being higher when compared to non‐biofilm‐producing isolates.

**TABLE 4:** MIC_50_ and MIC_90_ values of antimicrobial agents against biofilm‐producing *A. baumannii* and non‐biofilm‐producing *A. baumannii* isolates.

Antimicrobial agent (s)	Minimum inhibitory concentrations (μg/mL)				
	MIC range	Biofilm‐producing *A. baumannii* (*n* = 16)		Non‐biofilm‐producing *A. baumannii* (*n* = 22)	
		MIC_50_	MIC_90_	MIC_50_	MIC_90_
Imipenem	0.125 to >2	1	1	1	1
Meropenem	0.125 to >2	1	1	1	1
Ampicillin‐sulbactam	1 to >256	8	128	4	64
Cefoperazone‐sulbactam	0.5 to >256	>256	>256	128	>256
Piperacillin‐tazobactam	1/4 to >256/4	>256/4	>256/4	64/4	>256/4
Ceftriaxone	4 to >256	>256	>256	>256	>256
Cefotaxime	4 to >256	>256	>256	>256	>256
Ceftazidime	1 to >256	>256	>256	128	>256
Gentamicin	4 to >256	>256	>256	>256	>256
Amikacin	4 to >256	>256	>256	>256	>256
Ciprofloxacin	0.5 to >32	>32	>32	16	>32
Levofloxacin	0.5 to >32	>32	>32	16	>32
Trimethoprim‐sulfamethoxazole	1/38 to >4/76	>4/76	>4/76	>4/76	>4/76
Colistin	0.125 to 2	1	1	1	1
Tigecycline	0.125 to 2	1	1	1	1

### 3.3 Outcomes and factors influencing mortality

**FIGURE 1:**
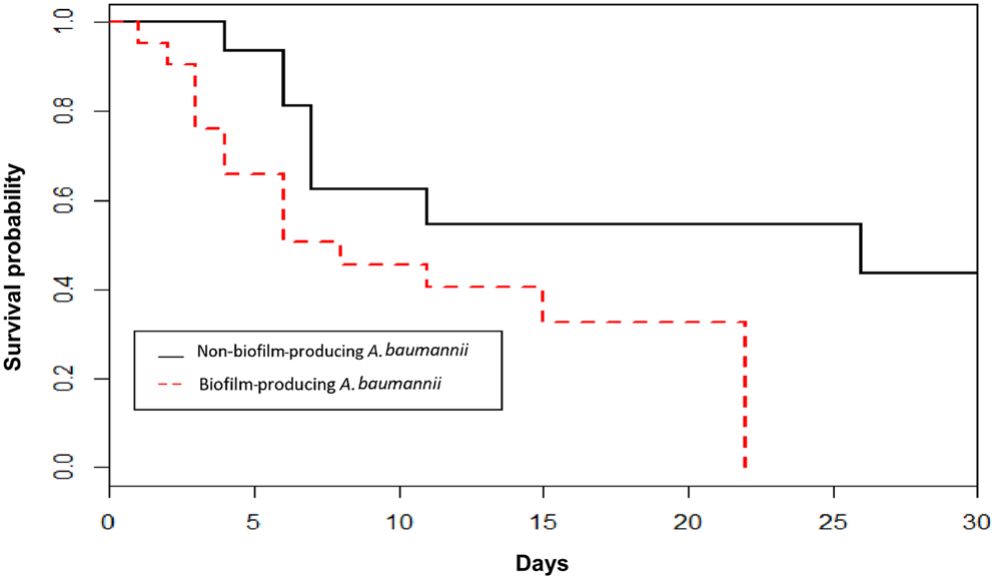
Kaplan–Meier survival curves at 30 days of the patients infected with biofilm‐producing and non‐biofilm‐producing *A. baumannii* isolates.

Comparisons of the outcomes between the patients diagnosed with VAP due to biofilm‐producing *A. baumannii* and those patients diagnosed with VAP due to non‐biofilm‐producing *A. baumannii* are demonstrated in Table 5. Overall, the patients infected with non‐biofilm‐producing *A. baumannii* had more complimentary outcomes, including the length of stay after the start of infection, mortality, hospital costs, and than those infected with biofilm‐producing *A. baumannii*, although only the differences in length of stay after the start of an infection and all types of hospital costs achieved statistical significance. Factors significantly influencing hospital mortality included APACHE II score, concomitant bacteremia, and inappropriate empirical antimicrobial therapy (Table 6). The survival rate of patients diagnosed with VAP due to biofilm‐producing *A. baumannii* was lower than in those with VAP due to non‐biofilm‐producing *A. baumannii* (*p* = 0.035, log‐rank test), as shown in Figure 1. The survival analysis with the Cox Proportional Hazards model demonstrated that the factors affected in‐hospital mortality were APACHE II score (Heart rate [HR], 1.18; 95% CI, 1.02 to 1.56; *p* = 0.009), concomitant bacteremia (HR, 1.31; 95% CI, 1.15 to 1.99; *p* = 0.007) and inappropriate empirical antimicrobial treatment (HR, 1.22; 95% CI, 1.05 to 1.77; *p* = 0.006), while infection with biofilm‐producing *A. baumannii* had only a borderline association with in‐hospital mortality (HR, 1.21; 95% CI, 0.98 to 1.43; *p* = 0.059).

**TABLE 5:** Comparisons of outcomes between the patients with ventilator‐associated pneumonia due to biofilm‐producing *A. baumannii* and those with ventilator‐associated pneumonia due to non‐biofilm‐producing *A. baumannii* .

Outcomes	Patients with ventilator‐associated pneumonia due to		*p‐*value
	Biofilm‐producing *A. baumannii* (*n* = 16)	Non‐biofilm‐producing *A. baumannii* (*n* = 22)	
Mortality, no. (%) of patients			
In‐hospital	9 (56%)	8 (36%)	0.223
7‐day	6 (37%)	6 (27%)	0.502
14‐day	7 (44%)	7 (32%)	0.450
30‐day	9 (56%)	8 (36%)	0.223
Median [IQR] of length of hospital stay after infection (days)	56 [29, 71]	42 [22, 68]	<0.001
Median [IQR] of cost (baht)			
Total hospital	90 452 [76 437–109 892]	56 436 [40 894–71 657]	<0.001
Antimicrobial	19 233 [13 587–30 453]	12 098 [9876–24 333]	<0.001
Non‐antimicrobial	71 200 [56 003–82 576]	48 554 [38 910–62 430]	<0.001

Abbreviation: IQR, interquartile range.

**TABLE 6:** Factors influencing in‐hospital mortality among the 38 patients with ventilator‐associated pneumonia due to *A. baumannii* .

Parameter	Values (%)		Crude OR (95% CI)	Adjusted OR (95% CI)	*p‐*value
	Survivors (*N* = 21)	Non‐survivors (*N* = 17)			
Median [IQR] of patient's age (years)	61 [48, 72]	66 [50, 75]	1.25 [1.05, 2.12]	1.11 [0.89, 2.04]	0.071
Male sex	12 (57%)	10 (59%)	1.07 (0.29, 3.91)	NI	NI
Comorbidities	20 (95%)	16 (94%)	0.80 (0.04, 13.81)	NI	NI
Emergency indication for admission	14 (67%)	16 (94%)	8.00 (0.87, 73.27)	5.23 (0.75, 29.12)	0.091
Median [IQR] of APACHE II score	15 [12, 18]	23 [18, 25]	1.38 [1.15, 3.36]	1.09 [1.01, 2.12]	0.048
Initial admission to intensive care unit	13 (62%)	15 (88%)	4.62 (0.82, 25.83)	3.25 (0.96, 16.88)	0.087
Retention of medical devices (including mechanical ventilator)	20 (95%)	15 (88%)	0.38 (0.03, 4.53)	NI	NI
Concomitant bacteremia	6 (29%)	12 (71%)	6.00 (1.47, 24.54)	2.51 (1.12, 19.04)	0.031
Inappropriate empirical antimicrobial therapy	5 (24%)	10 (59%)	4.76 (1.14, 20.00)	1.75 (1.09, 8.33)	0.046
Infection due to carbapenem‐resistant *A. baumannii*	5 (24%)	15 (88%)	24.01 (4.03, 43.99)	15.01 (3.62, 29.23)	<0.001
Infection due to biofilm‐producing *A. baumannii*	7 (33%)	9 (53%)	2.25 (0.60, 8.38)	1.36 (0.86, 4.55)	0.221

Abbreviations: APACHE II, Acute Physiology and Chronic Health Evaluation; CI, confidence interval; IQR, interquartile range; NI, not included to this model (*p*‐value > 9.2); OR, odds ratio.

## 4 DISCUSSION

Ventilator‐associated pneumonia (VAP) caused by *A. baumannii*, especially multidrug‐resistant (MDR) isolates become a public health concern with high morbidity and mortality rates. Importantly, biofilm formation by these pathogens is one of the most virulence factors that threaten the clinicians to treat the patients. Because the biofilm can prevent the penetration of the antimicrobial agents into the bacterial cells and contribute to the development of antimicrobial resistance.^14^, ^21^

Our study found that VAP due to biofilm‐producing *A. baumannii* had more unfavorable outcomes than those due to non‐biofilm‐producing *A. baumannii*. The biofilm‐producing isolates were significantly associated with the emergence of bacteremia and several antimicrobial resistances. These two properties together with a high initial APACHE II score influenced the in‐hospital mortality of the studied patients. Correlations between the extent of biofilm formation of the fungus and the emergence of bloodstream infections have been well established in infections due to *Candida albicans*,^22^ but data on similar associations with *A. baumannii* is scanty. One study found that sputum isolates of *A. baumannii* formed significantly more robust biofilm than blood isolates from the same patient.^23^ Our study exhibited that concomitant bacteremia was more common among patients with VAP due to biofilm‐producing *A. baumannii* than those with VAP due to non‐biofilm‐producing isolates. These findings can be explained by considering a previous in vitro study that found serum resistance by biofilm formation against complement activity of the human immune system.^23^ Additionally, whole genome sequencing analysis showed the presence of the genes responsible for biofilm formation, serum resistance, other virulence‐associated genes, and various antimicrobial resistance genes among *A. baumannii* isolates causing bacteremia or pneumonia.^17^, ^24^, ^25^

The association between biofilm and antibiotic resistance in nosocomial pathogens has been reported in previous studies.^26^, ^27^, ^28^, ^29^ Vatan et al. demonstrated that antibiotic use within the last 3 months was one of the significant factors related to biofilm, which is a major concern due to its role in antimicrobial resistance. Moreover, a significant positive correlation was seen between biofilm‐producing bacteria, especially Gram‐negative strains, and extensive drug resistance.^29^ In concordance with the study by Baidya et al., they also showed a high prevalence of multidrug resistance in biofilm‐producing Gram‐negative bacteria, but no significant relationship between biofilm formation and multidrug resistance was observed. However, there was a significant association between biofilm formation and piperacillin/tazobactam resistance in *Pseudomonas aeruginosa*.^28^ It is not surprising that we found a borderline association between biofilm formation and antimicrobial resistance. This can be generally explained by understanding the structure and functions of biofilm, particularly the quorum‐sensing system which regulates the genetic expression profile for antimicrobial resistance.^30^ It is interesting that the susceptibilities of the aminoglycosides (amikacin and gentamycin) in our study seemed relatively favorable compared to other antimicrobial agents, which were also found in previous studies.^30^ This can be explained by noting the high genetic barrier for aminoglycoside‐modifying enzymes that is the most common mechanism of antimicrobial resistance of these agents,^31^ and the possible high penetration of aminoglycosides in the biofilms that can interfere with the quorum‐sensing system.^30^ The biofilm‐producing isolates in our study exhibited relatively higher rates of carbapenem resistance than the non‐biofilm‐producing isolates. This finding is in contrast to previous studies conducted in patients with VAP due to *A. baumannii*, which can probably be explained by noting that other attributes can confer antimicrobial resistance in bacteria, allowing them to survive within a biofilm.^23^, ^32^

For the relationship between the biofilm and mortality, a previous study demonstrated that while patients infected with biofilm‐producing isolates exhibited higher mortality rates compared to those infected with non‐biofilm‐producing isolates, the crude mortality among VAP patients infected by these types of isolates did not show statistically significant differences.^26^ In our study, we found higher mortality in patients infected with non‐biofilm‐producing isolates than those infected with biofilm‐producing isolates, but the difference did not achieve significance. The biofilm formation showed only borderline associations with mortality rates with both multivariate analysis and survival analysis with the Cox Proportional Hazards model. This can be explained by noting that biofilm formation was only associated with other risk factors influencing mortality such as the emergence of bacteremia and poor antimicrobial susceptibility. The significant association between bacteremia and mortality can be also found in the patients infected with other Gram‐negative bacteria like *Stenotrophomonas maltophilia* clinical isolates.^33^ Thus, the impact of biofilm formation on the latter group is unclear and could have had an impact on our results. Additionally, our study focused on only VAP, and our results were potentially affected by biofilm formation in respiratory devices.

Some limitations in our study should be described. First, the retrospective nature of the study did not allow us to detect various factors possibly influencing mortality such as the indications for antimicrobial selection and overall treatment approaches. Second, the study populations in our study were relatively small, thus the non‐significant mortality rate difference between biofilm‐producing and non‐biofilm‐producing *A. baumannii* may have been due to the small sample size. Third, this study was conducted in a university hospital located in a single place, and the findings may not apply to other areas. Fourth, our study did not examine possible genetic influences to explain the phenotypic findings of biofilm formation and antimicrobial resistance. Last, this study investigated only isolates from sputum in patients with VAP and the emergence of bacteremia. Despite the above limitations, our study confirms the importance of biofilm formation on the survival of patients with VAP due to *A. baumannii*. Further investigations on how to manage biofilm‐producing *A. baumannii* are needed.

## AUTHOR CONTRIBUTIONS

Arnon Chukamnerd and Niwat Saipetch contributed to methodology, formal analysis, investigation, visualization, and writing—original draft. Kamonnut Singkhamanan, Natnicha Ingviya, and Nawaporn Assanangkornchai contributed to methodology, resources, and writing—review and editing. Komwit Surachat contributed to writing—review and editing. Sarunyou Chusri contributed to conceptualization, resources, data curation, writing—review and editing, supervision, project administration, and funding acquisition. All authors have read and approved the final manuscript.

## CONFLICT OF INTEREST STATEMENT

The authors declare no conflict of interest in this work.

## ETHICS STATEMENT

This study was approved by the Institutional Review Board of Human Research Ethics Unit (HREU), Faculty of Medicine, Prince of Songkla University, Songkhla, Thailand (ethical reference number: 61‐155‐14‐4). The ethics committees were allowed to waive the patient consent, according to the retrospective study.

## Data Availability

The data that support the findings of this study are available from the corresponding author upon reasonable request.
